# Cannabis Liberalization and Adolescent Cannabis Use: A Cross-National Study in 38 Countries

**DOI:** 10.1371/journal.pone.0143562

**Published:** 2015-11-25

**Authors:** Yuyan Shi, Michela Lenzi, Ruopeng An

**Affiliations:** 1 Department of Family Medicine and Public Health, University of California San Diego, La Jolla, California, United States of America; 2 Department of Developmental and Social Psychology, University of Padova, Padua, Italy; 3 Department of Kinesiology and Community Health, University of Illinois at Urbana-Champaign, Champaign, Illinois, United States of America; University of Newcastle, Australia, AUSTRALIA

## Abstract

**Aims:**

To assess the associations between types of cannabis control policies at country level and prevalence of adolescent cannabis use.

**Setting, Participants and Design:**

Multilevel logistic regressions were performed on 172,894 adolescents 15 year of age who participated in the 2001/2002, 2005/2006, or 2009/2010 cross-sectional Health Behaviour in School-Aged Children (HBSC) survey in 38 European and North American countries.

**Measures:**

Self-reported cannabis use status was classified into ever use in life time, use in past year, and regular use. Country-level cannabis control policies were categorized into a dichotomous measure (whether or not liberalized) as well as 4 detailed types (full prohibition, depenalization, decriminalization, and partial prohibition). Control variables included individual-level sociodemographic characteristics and country-level economic characteristics.

**Findings:**

Considerable intra-class correlations (.15-.19) were found at country level. With respect to the dichotomized cannabis control policy, adolescents were more likely to ever use cannabis (odds ratio (OR) = 1.10, p = .001), use in past year (OR = 1.09, p = .007), and use regularly (OR = 1.26, p = .004). Although boys were substantially more likely to use cannabis, the correlation between cannabis liberalization and cannabis use was smaller in boys than in girls. With respect to detailed types of policies, depenalization was associated with higher odds of past-year use (OR = 1.14, p = .013) and regular use (OR = 1.23, p = .038), and partial prohibition was associated with higher odds of regular use (OR = 2.39, p = .016). The correlation between cannabis liberalization and regular use was only significant after the policy had been introduced for more than 5 years.

**Conclusions:**

Cannabis liberalization with depenalization and partial prohibition policies was associated with higher levels of regular cannabis use among adolescents. The correlations were heterogeneous between genders and between short- and long-terms.

## Introduction

Cannabis use, especially regular use, is associated with adverse health consequences including dependence symptoms, respiratory and cardiovascular diseases, impaired psychosocial development, psychotic outcomes, and traffic fatalities. [1–4] Cannabis remains the most commonly used illicit drug in the world, with estimated 125–203 million current users in 2009. [5] The prevalence is high among adolescents. In Europe and North America, 16.4% boys and 12.0% girls 15 years of age used cannabis in past year, and 3.0% boys and 0.8% girls used regularly. [6,7]

Following provisions of 1925 “International Opium Convention” in Geneva, non-medical cannabis use became criminal offence in all countries participating in the Geneva Convention. The 1961 Single Convention on Narcotic Drugs further limited the possession, use, trade in, distribution, and production of cannabis drugs even for medical use purpose. [8,9] The strong law enforcement after the 1961 Convention led to a substantially increased number of cannabis-related arrests in European and North American countries, predominantly among younger population. [9] During the past half century, however, the punitive approach has been under considerable scrutiny. There were longstanding debates around the harmfulness of cannabis use and the adverse social and economic consequences of drug enforcement. [9–14]

From the late twentieth century, many countries had policy reforms to liberalize the traditional regime of criminal prohibition of cannabis. Some examples include Australia, Canada, Netherlands, United States, Uruguay, and many other countries in Europe. While some jurisdictions such as Uruguay and Washington, Colorado in the United States also regulated production and distribution very recently, [15,16] the liberalization reforms primarily focused on possession and use of small quantities of cannabis. Room et al. [9] provided a detailed review of the evolution of cannabis liberalization in the world and proposed categorization of alternative cannabis control policies. Considering the heterogeneities in the presence of criminal sanctions, roles of judiciary and police, forms of penalties, levels of law enforcement, and target population, cannabis control regimes can be categorized into 4 types, including full prohibition, depenalization, decriminalization, and partial prohibition. [9,17] The latter 3 regimes represent existing forms of cannabis liberalization policies.

Along with the development and implementation of cannabis liberalization, there have been considerable concerns about the increased cannabis use prevalence induced by increased access, more favorable social norms, and reduced penalties and cost. [9,18,19] Nonetheless, the empirical research on cannabis use associated with different types of cannabis control policies is surprisingly limited. There is only initial evidence suggesting an increasing trend in the prevalence of cannabis use and its association with the adoption of cannabis liberalization policies within countries such as United States and Australia. [5,20–23] Some other studies, however, did not find such a relationship. [19,24–30]

The onset of cannabis use typically occurs among adolescents, who are particularly vulnerable to the development of substance use disorders and other harms. [31,32] From public health and cost-benefit perspectives, preventing cannabis use in early life is more desirable because it yields greater health and economic benefits and requires less costs and efforts than inducing treatment in later life. However, even fewer empirical studies evaluated the association between cannabis use and cannabis control policies in adolescent population, [18,19,30] and most of them have been restricted to within-country analysis that focused on a single type of policy.

The correlations between cannabis control policies and cannabis use may be dynamic. The impacts of the policy introduction could be dependent upon the length of time the policy has taken effect. For example, one may argue that cannabis liberalization may lead to lower perceived risks and easier access in the short term, whereas it takes longer time for people to change initiation and cessation behaviors in response to the changes in the environment. One may also argue that curiosity-driven cannabis use will be reduced after policies have passed for a long time, such that the effects of liberalization policies are larger in a short term. Very limited empirical data have been provided to support either argument. [20]

This study aimed to examine cannabis control policies in relation to cannabis use among adolescent population in 38 European and North American countries. Specifically, we reviewed existing literature to characterize types of cannabis control policies in each country, and conducted multilevel models to statistically assess the associations of cannabis use to the types of cannabis control policies. The heterogeneities in gender and duration of policy implementations were also examined.

## Methods

### Sample

Health Behaviour in School-Aged Children Study (HBSC) data were used in this study. HBSC is a repeated cross-sectional international survey on country-representative adolescents in 3 age groups: 11-, 13-, and 15-year-olds. Since 1983, it collects comparable information on health, health behaviors, and social environment every four years in 20–40 participating countries across Europe and North America. Two-stage cluster random sampling is used to sample schools then classes. The sample size in each country and age group is sufficient for prevalence estimate with a 95% confidence interval of ±3% and a design factor of 1.2. [33]

Participating countries adhered to international research protocol to ensure consistency in sampling, questionnaires, and survey implementation, and followed regulations on ethics and data protection in respective institutions and countries. Internationally standardized questionnaires were voluntarily completed in classroom setting with paper or computer-assisted format. The response rates at school level differ across countries, ranging from 44% to 92%. Data can be accessed at http://www.hbsc.org/data by submitting data access forms. Detailed information on HBSC data can be found elsewhere. [33,34]

Our study sample restricted to 15-year-old adolescents in the three most recent surveys in 2001/2002, 2005/2006, and 2009/2010, the only age group with cannabis questions available since 2001. A total of 172,894 adolescents from 38 countries provided complete information on cannabis use and entered statistical analyses. Those who did not respond to cannabis use questions (N = 7010 or 4%) were slightly more likely to be boys and with lower family affluence.

### Cannabis use

Adolescents reported the frequency of cannabis use in life time as well as in the past 12 months with 7 scales: never, 1–2 times, 3–5 times, 6–9 times, 10–19 times, 20–39 times, and 40 or more. Accordingly, we defined 3 dichotomous outcomes to measure adolescents’ cannabis use status: 1) ever used cannabis in life time; 2) used cannabis in past year; and 3) used cannabis regularly. Following previous literature, [6,35] regular cannabis use was identified if adolescents reported using cannabis 40 times or more in life time.

### Cannabis control policy

We primarily relied on Room et al., [9] and European Monitoring Centre for Drugs and Drug Addiction (EMCDDA) to characterize country-level cannabis control policies. We adopted categorization proposed in Room et al., [9] which identified 4 models of cannabis control at the country level: 1) full prohibition, or the traditional criminal prohibition regime, 2) depenalization, or prohibition with cautioning or diversion; 3) decriminalization, or prohibition with civil penalties, and 4) partial prohibition, including ‘*De facto’* and ‘*De jure’* legalization. With the traditional full prohibition policy, cannabis use is criminal offense and subject to criminal control. Cannabis use remains criminal offense with depenalization policy, but the severity of punitive consequences is reduced by various alternatives. With decriminalization policy, cannabis use is regulated by non-criminal statues or interventions and criminal processing and punishment are replaced with civil or administrative sanctions. Partial prohibition policy selectively enforces criminal laws associated with cannabis use, or allows cannabis use at select spaces or populations. [9] Depenalization, decriminalization, and partial prohibition represent alternative approaches to liberalize cannabis use. Thus we created a binary indicator (full prohibition vs. depenalization, decriminalization and partial prohibition) to distinguish these liberalization policies from the traditional full prohibition. To allow differential correlations between cannabis use and cannabis liberalization by duration of policy implementation, we also created short-term (0–5 years), mid-term (5–10 years), and long-term (more than 10 years) indicators for the length of time the policy had taken effect by the interview year.

While most countries regulate cannabis use by the central government, some regional jurisdictions (state, county, or city) within a country may have authority to enact and enforce their own cannabis control policies. As a result, cannabis control maybe liberalized in some areas in a country, but not in others. [9,36] The most noticeable example is United States, in which some states have legalized cannabis use for medical and/or recreational purposes but cannabis remains illegal at federal level. Because HBSC data did not provide detailed geographic identifiers other than country, adolescents’ exposure to the regional policies were unfortunately not able to be identified. We defined the United States as liberalized in the main analysis even though cannabis use was only depenalized or partially prohibited in part of the country, [9] for the rationale that liberalization in some jurisdictions may have influenced social norms, access, and price at the national level. We performed additional analysis to test the sensitivity of this arrangement.

### Individual-level characteristics

We considered the following socio-economic individual characteristics that were suggested correlates of cannabis use among adolescents. [6,35,37–42] Variables were selected a priori. Family structure was measured with 2 items: whether or not living with both parents, and number of siblings. Perceived social support from family was assessed by the difficulty of communication with parents: “how easy is it for you to talk to the following persons about things that really bother you”. Responses regarding communication with father and mother were separately rated on a 4-point scale (1 = very easy to 4 = very difficult) and averaged, with higher value indicating larger difficulty to communicate with parents. [41] Three measures described social support from peers: difficulty of communication with friends, number of friends, and time spent with friends. Same algorithm as difficulty of communication with parents was applied to achieve a summary scale for difficulty of communication with best friend, friend(s) of the same sex, and friend(s) of opposite sex. [41] Two items were used to measure time spent with friends: the number of days a week that adolescents spend time with friends after school and the number of evenings they spend out with friends were averaged, with higher value representing larger frequency of contacts with friends. [41] The validated measure of psychological complaints [41,43,44] was derived from 5 items: “in the last 6 months: how often have you had the following: 1) feeling low, 2) irritability or bad temper; 3) feeling nervous; 4) difficulties in getting to sleep; and 5) feeling dizzy”. Responses to each item were rated with a 5-point scale (1 = about every day to 5 = rarely or never). Reversed responses were averaged, with higher value suggesting higher level of psychological complaints. The validated family affluence (FAS) [40] is a summary scale of household material conditions. It was computed by summing up responses to questions on possession of car, own bedroom, number of computers, and frequency of vacations during the past year. The summary scale ranges between 0 and 9, with scale 0–3 representing low affluence, 4–5 representing medium affluence, and 6–9 representing high affluence.

### Country-level characteristics

Estimates on GDP per capita in the survey year were obtained from the World Bank [45]. It is the aggregated gross values added by all resident producers in the economy divided by midyear population of the country [45]. We converted monetary values to 2010 US dollars and grouped into three tertiles to represent lower, medium, and higher income countries.

### Statistical analysis

We combined 2001/2002, 2005/2006, and 2009/2010 HBSC data in the analyses. Individual-level cannabis use measures were summarized by country and gender. As the data structure is two-level with students nested in countries, multilevel logistic random intercept model was performed to assess the associations between cannabis use and cannabis control policies. Two models were conducted with cannabis use as the outcome variable and cannabis control policy as the explanatory variable. Specifically, the first model considered the binary indicator for the broad type of cannabis control policies (whether or not liberalized) as the primary explanatory variable of interest; and the second model instead entered detailed types of cannabis control policies (fully prohibition, depenalization, decriminalization, or partial prohibition). Preliminary analyses and prior literature suggested heterogeneity in distributions of cannabis use and differential responses to drug policies between boys and girls [6,35,39], we therefore added interaction terms in the two models to allow different levels of policy responses by gender. Individual sociodemographic characteristics, country-level economic characteristic, and survey year indicators were also added to the regressions. As a supplementary analysis, we replaced the cannabis policy indicator with the indicators of policy durations to test the differential correlations in the short- and long-terms.

Stata 12 (StataCorp LP) was used for statistical analyses. Adjusted odds ratios and 95% confidence intervals were reported. We also reported intraclass correlation (ICC) that describes the degree to which adolescents’ cannabis use in the same country resembles each other. All reported statistics were weighted to be country-representative, unless noted otherwise. Institutional Review Board approval is not required as this study used secondary data.

## Results

### Sample and country characteristics

Prevalence of cannabis use by country and gender was reported in Table 1. A total of 83,294 boys and 89,600 girls were included in the study. The weighted prevalence of ever use, past-year use, and regular use during 2001–2010 in 38 countries was 19.85%, 15.56%, and 3.32%, respectively. There were substantial differences in the prevalence across countries. In general, boys had higher rates of cannabis use compared to girls.

**Table 1:** Summary of cannabis use prevalence by country and gender, HBSC 2001–2010 (N = 172,894).

Country	Total number of students, unweighted N	Boys, %	Ever used cannabis, %			Used cannabis in past year, %			Used cannabis regularly, %		
			Total	Boys	Girls	Total	Boys	Girls	Total	Boys	Girls
Austria	4,354	47.52	13.73	14.60	12.95	10.21	11.66	8.90	1.87	2.67	1.14
Armenia	814	42.87	4.18	8.88	0.65	3.63	7.64	0.70	0.25	0.58	0.00
Belgium	8,904	50.69	23.28	26.15	20.34	18.85	21.40	16.22	4.38	6.21	2.51
Bulgaria	1,677	47.64	19.32	20.65	18.11	13.30	14.65	12.08	2.27	3.38	1.26
Canada	8,405	46.29	35.24	36.14	34.46	29.85	30.41	29.38	8.91	10.88	7.22
Croatia	5,405	46.75	14.47	17.25	12.02	11.35	13.42	9.53	1.98	2.82	1.25
Czech Republic	4,725	49.04	28.89	31.42	26.45	22.61	24.28	21.02	3.80	4.89	2.75
Denmark	3,990	47.57	18.95	21.50	16.63	14.87	16.98	12.96	1.79	2.76	0.91
Estonia	4,209	48.99	22.07	27.74	16.63	16.00	19.91	12.26	1.52	2.53	0.56
Finland	5,327	47.61	9.24	10.76	7.85	7.18	8.26	6.21	0.96	1.54	0.43
France	6,592	49.61	28.40	31.44	25.41	24.18	26.46	21.95	5.26	7.32	3.23
Germany	5,681	47.69	17.26	20.37	14.43	12.52	15.06	10.23	2.68	3.84	1.62
Greece	4,122	47.83	5.68	8.59	3.02	4.56	6.99	2.34	0.96	1.71	0.27
Greenland	870	44.83	27.93	29.49	26.67	18.88	20.11	17.90	2.82	3.67	2.12
Hungary	4,067	42.81	13.86	17.13	11.42	10.44	12.79	8.69	1.21	2.38	0.34
Iceland	5,502	50.62	9.27	12.14	6.33	6.89	9.17	4.57	1.46	2.02	0.88
Ireland	4,232	51.61	19.99	23.26	16.50	16.42	19.42	13.26	4.73	6.49	2.85
Israel	4,473	43.05	6.26	9.59	3.74	5.43	8.19	3.36	0.90	1.64	0.34
Italy	3,797	48.35	20.78	24.84	16.98	18.13	21.35	15.11	2.77	3.49	2.09
Latvia	3,620	45.25	20.36	25.58	16.04	16.16	20.93	12.08	1.30	2.63	0.20
Lithuania	5,474	51.26	14.72	19.92	9.26	9.47	12.96	5.84	0.86	1.50	0.19
Luxembourg	2,860	50.91	21.50	24.11	18.80	17.11	19.40	14.75	3.66	4.98	2.29
Malta	956	48.33	8.89	11.04	6.88	7.30	8.22	6.44	1.79	2.61	1.02
Netherlands	4,070	50.18	23.47	25.60	21.31	19.28	21.34	17.21	4.42	5.92	2.90
Norway	1,313	52.78	5.79	7.22	4.19	4.40	6.02	2.60	0.92	1.59	0.16
Poland	5,760	47.81	18.49	24.51	12.97	14.17	19.48	9.32	1.91	3.31	0.63
Portugal	3,710	44.58	13.58	17.29	10.60	10.93	14.38	8.17	2.20	3.16	1.43
Romania	3,491	45.57	6.36	9.81	3.47	4.31	6.93	2.14	0.26	0.44	0.11
Russia	6,425	45.37	13.42	17.19	10.28	8.39	11.16	6.09	1.12	1.96	0.43
Slovakia	2,883	47.83	17.52	21.32	14.03	12.58	15.43	9.98	1.18	2.11	0.33
Slovenia	4,336	50.05	22.81	26.64	18.98	17.64	20.52	14.79	4.34	5.50	3.19
Spain	6,111	47.71	31.64	31.96	31.34	25.72	25.68	25.77	6.65	8.16	5.26
Sweden	1,212	49.92	7.01	7.11	6.92	4.69	4.87	4.51	0.91	0.99	0.83
Switzerland	4,952	49.90	34.87	39.50	30.27	28.14	31.08	25.24	7.32	9.80	4.85
Ukraine	5,137	48.08	16.86	24.69	9.60	9.60	14.93	4.76	1.05	1.91	0.26
Macedonia	4,739	50.41	3.59	4.60	2.55	2.76	3.62	1.89	0.32	0.54	0.09
United Kingdom	14,128	49.33	28.42	29.81	27.07	22.68	23.77	21.63	5.78	7.66	3.96
United States	4,571	49.07	31.63	34.20	29.17	25.57	27.78	23.46	8.68	10.94	6.50
**Total**	**172, 894**	**48.24**	**19.85**	**22.92**	**16.99**	**15.56**	**17.96**	**13.33**	**3.32**	**4.59**	**2.14**

Nine countries have 1 or 2 survey data missing because they opted not to participate in the HBSC survey or administer cannabis questions in that year. The missing data are: Armenia (2001/2002, 2005/2006), Bulgaria (2001/2002, 2009/2010), Iceland (2001/2002), Luxembourg (2001/2002), Malta (2009/2010), Norway (2001/2002, 2005/2006), Romania (2001/2002), Slovakia (2001/2002), and Sweden (2005/2006, 2009/2010).

Country-level variables were summarized in Table 2. Out of the 38 countries, we identified 20 countries that have liberalized cannabis use to some extent during the study period. Among these 20 countries, 4 adopted policies to depenalize cannabis use; 11 decriminalized cannabis use; and 7 implemented partial prohibition policies.

**Table 2:** Summary of country-level variables.

	Per-capita GDP, in 2010 US dollars [45]	Types of cannabis use control policy as of 2010 and effective date [9,47]
Austria	$40,665	Partial Prohibition, 1998
Armenia	$3,125	Decriminalization, 2008
Belgium	$38,889	Decriminalization, 2003
Bulgaria	$4,720	Full Prohibition
Canada	$39,987	Depenalization, 1996 & Partial Prohibition, 2001
Croatia	$11,031	Full Prohibition
Czech Republic	$15,277	Decriminalization, 2010
Denmark	$51,353	Decriminalization, 2004
Estonia	$11,547	Decriminalization, 2002
Finland	$41,027	Full Prohibition
France	$36,516	Depenalization, 1999
Germany	$37,165	Partial Prohibition, 1994
Greece	$23,403	Full Prohibition
Greenland	$26,856	Full Prohibition
Hungary	$11,078	Full Prohibition
Iceland	$51,247	Full Prohibition
Ireland	$48,436	Full Prohibition
Israel	$25,312	Partial Prohibition, 1992
Italy	$32,948	Decriminalization, 1990
Latvia	$9,336	Decriminalization, 1999
Lithuania	$9,982	Decriminalization, 1998 (ended 2003)
Luxembourg	$99,165	Decriminalization, 2001
Malta	$15,045	Full Prohibition
Netherlands	$44,224	Partial Prohibition, 1976
Norway	$86,096	Full Prohibition
Poland	$9,497	Full Prohibition
Portugal	$19,844	Decriminalization, 2001
Romania	$7,196	Full Prohibition
Russia	$7,019	Decriminalization, 2004
Slovakia	$15,335	Full Prohibition
Slovenia	$19,672	Full Prohibition
Spain	$27,364	Partial Prohibition, 1992
Sweden	$35,782	Full Prohibition
Switzerland	$62,077	Full Prohibition
Ukraine	$2,175	Full Prohibition
Macedonia	$3,486	Full Prohibition
United Kingdom	$39,444	Depenalization, 2004 (ended 2009)
United States	$48,237	Depenalization in some jurisdictions, 1973 & Partial Prohibition in some jurisdictions, 1996

Per-capita GDP was converted to 2010 US dollars and averaged across survey years.

**Fig 1:**
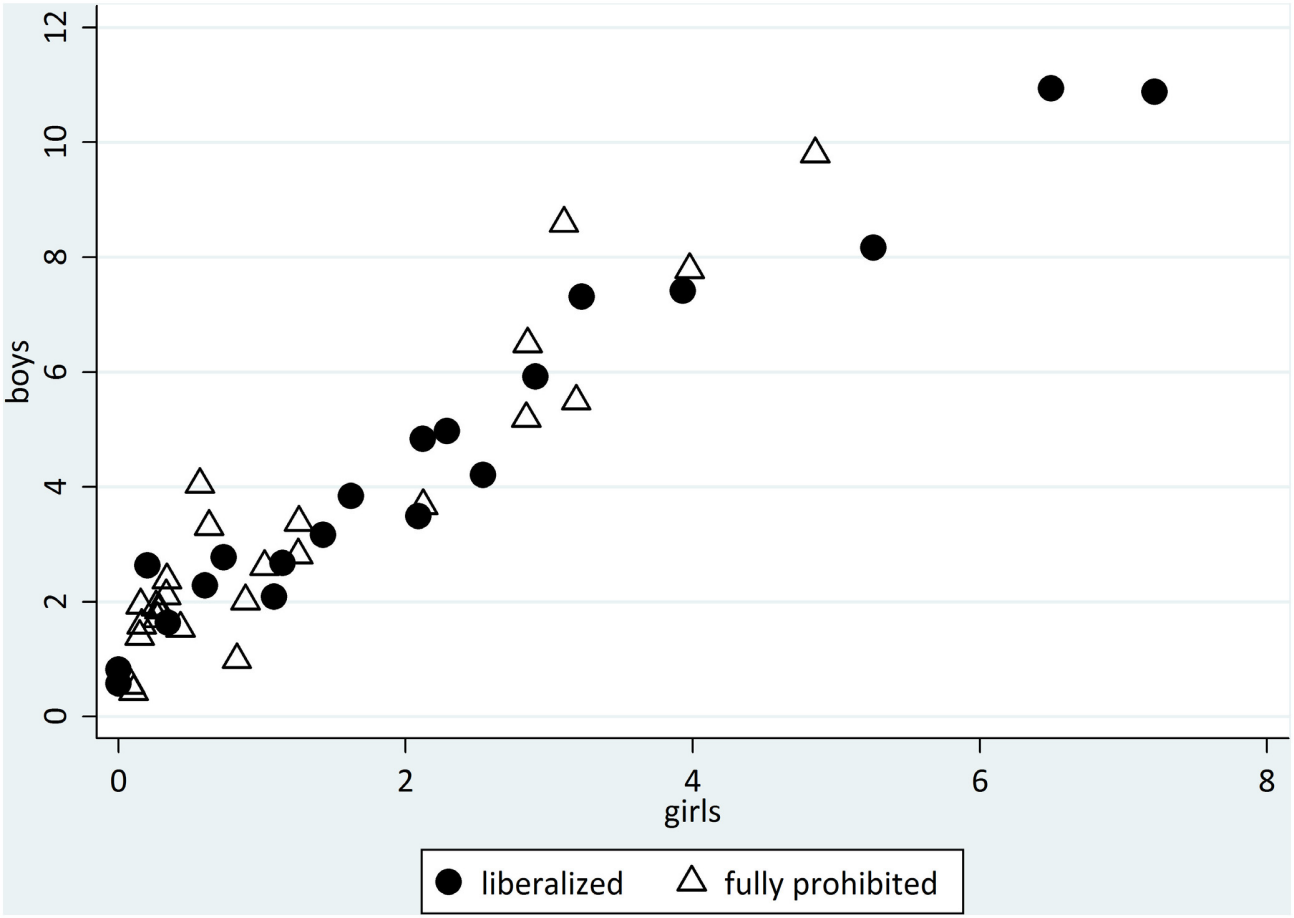
Scatter plot of prevalence of regular cannabis use, by gender and cannabis liberalization status (%). HBSC 2001–2010 (N = 172,894).

Prevalence of regular cannabis use was plotted against cannabis liberalization status (Fig 1). Overall, countries that fully prohibited cannabis use tended to concentrate at the lower left corner, where the prevalence of regular use in both boys and girls was smaller relative to countries in which cannabis use had been liberalized.

### Cannabis control policy and cannabis use

Multilevel logistic regressions were conducted to assess the associations between cannabis use and cannabis control policies. In the model with broad type of cannabis control policy as the primary explanatory variable (Table 3), adolescents were more likely to ever use cannabis (OR = 1.10, p = .001), use in past year (OR = 1.09, p = .007) and use regularly (OR = 1.23, p = .004) if they lived in countries that had liberalized cannabis use. Even though boys had a substantially higher prevalence of cannabis use, the interaction terms between cannabis liberalization and boys were significantly smaller than 1 for all three cannabis use measures (p < .001). This indicated that the correlation between cannabis use and cannabis liberalization was weaker in boys compared to girls.

**Table 3:** Multilevel logistic regressions for adolescent cannabis use status and broad type of cannabis control policies. HBSC 2001–2010 (N = 172,894).

	Ever used cannabis	Used cannabis in past year	Used cannabis regularly
**Individual-level variables**			
Boy	1.91***	1.86***	3.08***
Living with both parents	.63***	.63***	.55***
Number of siblings	1.00	.99	1.04***
Difficulty of communication with parents	1.34***	1.36***	1.30***
Difficulty of communication with friends	.72***	.72***	.69***
Number of friends	1.04***	1.03***	1.00
Time spent with friends	1.41***	1.41***	1.55***
Psychological complaints	1.48***	1.51***	1.63***
Family affluence = medium	1.10***	1.11***	.96
Family affluence = high	1.17***	1.22***	.96
**Country-level variables**			
Cannabis liberalization	1.10***	1.09**	1.23**
Cannabis liberalization*boy	.80***	.82***	.77***
Per-capita GDP level = second tertile	1.17*	1.03	1.59**
Per-capita GDP level = third tertile	1.38***	1.12	1.73***
Survey = 2005/2006	.76***	.69***	.70***
Survey = 2009/2010	.74***	.68***	.62***
**Country-level variation (ICC)**	.15	.15	.19

*p < .05,

**p < .01,

***p < .001

Table 4 reported the estimates on the detailed types of cannabis control policies. The odds of past-year cannabis use among adolescents living in countries with depenalization policies was 1.14 times (p = .013) as high as their counterparts in other countries. Depenalization and partial prohibition policies predicted higher level of regular cannabis use (OR = 1.23, p = .038; OR = 2.39, p = .016, respectively). The correlations between depenalization policy and cannabis use were significantly smaller in boys relative to girls (p < .001), so were the correlations between partial prohibition and cannabis use (p < .01).

**Table 4:** Multilevel logistic regressions for adolescent cannabis use status and detailed type of cannabis control policies. HBSC 2001–2010 (N = 172,894).

	Ever used cannabis	Used cannabis in past year	Used cannabis regularly
**Individual-level variables**			
Boy	1.87***	1.82***	3.08***
Living with both parents	.63***	.63***	.55***
Number of siblings	1.00	.99	1.04***
Difficulty of communication with parents	1.34***	1.36***	1.30***
Difficulty of communication with friends	.72***	.72***	.69***
Number of friends	1.03***	1.03***	1.00
Time spent with friends	1.41***	1.41***	1.55***
Psychological complaints	1.48***	1.51***	1.63***
Family affluence = medium	1.10***	1.10***	.95
Family affluence = high	1.17***	1.21***	.96
**Country-level variables**			
Cannabis depenalization	1.09	1.14*	1.23*
Cannabis decriminalization	1.01	.97	1.07
Cannabis partial prohibition	1.60	1.76	2.39**
Cannabis depenalization*boy	.77***	.76***	.77***
Cannabis decriminalization*boy	.98	1.02	.94
Cannabis partial prohibition*boy	.85***	.89**	.82**
Per-capita GDP level = second tertile	1.16*	1.02	1.54**
Per-capita GDP level = third tertile	1.37***	1.11	1.67***
Survey = 2005/2006	.76***	.69***	.70***
Survey = 2009/2010	.74***	.68***	.62***
**Country-level variation (ICC)**	.15	.15	.17

*p < .05,

**p < .01,

***p < .001

The intra-class variation was substantial in both models (ICC = .15-.19), suggesting that a large fraction of variance can be explained by country-level characteristics. A few individual-level socio-economic characteristics were consistently associated with cannabis use outcomes across models. For instance, adolescents living with both parents and having greater difficulty in communication with friends were less likely to use cannabis. Adolescents who had larger difficulty in communication with parents, spent more time with friends, and had higher level of psychological complaints had higher odds of using cannabis. Family affluence was associated with ever cannabis use and past year use, but not with regular use. The influences of country-level per-capita GDP measure were only significant in ever use and regular use models. Compared to survey 2001/2002, the prevalence of cannabis use was significantly smaller in survey 2005/2006 and 2009/2010.

The correlations between cannabis use and duration of policy implementation were reported in Table 5. Cannabis liberalization was significantly correlated with a higher odds of using cannabis regularly after the policy had been introduced for 5–10 years (OR = 1.27, p = .010) and more than 10 years (OR = 1.43, p = .002), whereas the correlation was not significant within 5 years of policy implementation. Duration of policy implementation had no discernable impacts on ever use or past-year use of cannabis.

**Table 5:** Multilevel logistic regressions for adolescent cannabis use status and length of time that cannabis liberalization policies took effect. HBSC 2001–2010 (N = 172,894).

	Ever used cannabis	Used cannabis in past year	Used cannabis regularly
Cannabis liberalized between 0–5 years	.94	.97	1.06
Cannabis liberalized between 5–10 years	1.08	1.05	1.27**
Cannabis liberalized more than 10 years	1.05	1.01	1.43**
**Country-level variation (ICC)**	.15	.15	.17

*p < .05,

**p < .01,

***p < .001

All models also included individual-level variables, per-capita GDP categories, and survey year fixed effects.

### Sensitivity analysis

The adolescents in the United States were removed from the study sample as a sensitivity check. The findings regarding the associations between cannabis control policy and cannabis use were qualitatively consistent with those estimated from the full sample (detailed results can be found at http://dx.doi.org/10.6084/m9.figshare.1598138).

## Discussion

Although cannabis use has been liberalized in many western countries, its association with cannabis use behaviors remains largely unknown. To our knowledge, this is the first study with a global perspective to examine country-level cannabis policy in relation to adolescent cannabis use. Drawing pooled cross-sectional data from over 170,000 adolescents in 38 European and American countries, we assessed whether and how types of cannabis control policies were correlated with ever use, past-year use, and regular use of cannabis.

We found that substantial variation in the prevalence of cannabis use can be attributed to the country-level characteristics. Overall, cannabis liberalization was associated with higher likelihood of ever use, past-year use, and regular use of cannabis. Significant positive correlations were found between cannabis depenalization and past-year and regular use, and between partial prohibition and regular use. Detailed types of cannabis control policies had no correlation with ever use of cannabis. Those who ever used cannabis but did not use in past year or use regularly were primarily discontinued users or experimental users. [42] The heterogeneities in the impacts of cannabis control policies highlighted the importance of making distinctions between different types of cannabis users.

Our study findings were supported by the demand theory that less strict laws and enforcement induce more drug use. Simons-Morton et al. [18] suggested no associations between country-level policies and cannabis use prevalence in the United States, Canada, and the Netherlands, which all have liberalized cannabis use with depenalization or partial prohibition policies. Their findings were consistent with the estimated associations between detailed types of policies and regular cannabis use in our study. Our findings were also supported by a few within-country studies, which showed a correlation between a single type of cannabis liberalization policy and increased cannabis use. [20–22] Some other within-country studies did not reveal such relationship. [19,24,25,28–30,46] It is worth noting that within-country studies are not comparable to cross-sectional and cross-national analyses like our study, because they restricted to a single type of policy and took advantage of regional variations in the timing of policy implementations within a country. Cross-country comparisons with sufficient observations before and after the policy implementation are warranted in future research.

Boys had a considerable higher level of cannabis use compared to girls, as documented in previous research. [6] However, the associations between cannabis use and cannabis control policies were in general smaller in boys. This finding was consistent with the prior literature that demonstrated differential associations between tobacco control policies and adolescent smoking behaviors by gender. [39] The mechanisms of the gender differences in association with cannabis control policies are still unclear. Future investigations are encouraged to consider and examine the heterogeneities in policy responses between genders.

This study revealed the correlations between regular use of cannabis and duration of cannabis control policies. Unlike Williams et al. [20] that found the impact of cannabis liberalization in Australia concentrated in the first 5 years following the policy introduction, our cross-national findings suggested a larger correlation between regular use and cannabis liberalization after the policy had taken effect for more than 5 years. The differential associations in the short- and long-terms underscore the importance of data collection in a long policy window, which will allow sufficient period of time to observe any changes in cannabis use behaviors in response to changes in the policy environment.

Several limitations of this study are noteworthy, some of which are common in other studies using cross-sectional data and cross-country comparisons. [6,37,39] First, this observational study assessed associations instead of causal relationships. The data could not infer whether the correlation was due to the influences of cannabis liberalization, pre-existing differences in prevalence or social norms, or other confounding factors at individual- or country level that were not controlled in the study. Second, the cannabis use measures are subject to self-report and recall bias as any drug use measures are in population surveys. Third, just like any other similar multi-country surveys, the geographic identifiers other than country are not provided in HBSC data. We were not able to examine cannabis control policies at regional level, which may differ from national measures. Some countries with prohibition policies may share boarders with countries that have liberalized cannabis use, thus individuals living close to boarders might be influenced by adjacent country’s legal environment. Nonetheless, this is less of a concern in this study because the mobility of the adolescents is presumably very limited. Fourth, liberalizing cannabis control can result in increased accessibility, lower price, reduced perceived harmfulness, and increased social approval. [9,19,22] We were not able to explore these mechanisms in association with cannabis prevalence due to data limitations. In addition, cannabis control reform is complicated. Although we adopted a detailed 4-point scale as well as a crude binary indicator to categorize country policies, they still may not account for subtle differences across countries. Particularly, the policies on the books may be deviated from the policies in action. Countries in the same policy category may vary considerably in terms of law enforcement levels in reality. Last, the sample size of countries are insufficient to support investigation on duration of detailed types of policies, and the study findings may not be generalizable to the whole adolescence.

## Conclusion

Cannabis control in many western countries has departed from the full prohibition regime towards liberalization, with various models adopted including depenalization, decriminalization, and partial prohibition. Despite the limitations, this study for the first time examined the associations between country-level cannabis control policies and cannabis use from a global perspective in the adolescent population, the vulnerable group at a high risk of drug initiation. Our study showed that the liberalization policy in general was associated with higher levels of cannabis use, and depenalization and partial-prohibition policies were particularly correlated with regular use. The correlations were heterogeneous between genders and between short- and long-terms. Efforts to prevent cannabis use among adolescents are recommended in countries that have embraced liberalization policies, with particular attention to gender differences and policy dynamics.
